# Normalizing the Microenvironment Overcomes Vessel Compression and Resistance to Nano‐immunotherapy in Breast Cancer Lung Metastasis

**DOI:** 10.1002/advs.202001917

**Published:** 2020-12-13

**Authors:** Fotios Mpekris, Myrofora Panagi, Chrysovalantis Voutouri, John D. Martin, Rekha Samuel, Shinichiro Takahashi, Naoto Gotohda, Toshiyuki Suzuki, Panagiotis Papageorgis, Philippos Demetriou, Chryso Pierides, Laura Koumas, Paul Costeas, Motohiro Kojima, Genichiro Ishii, Anastasia Constantinidou, Kazunori Kataoka, Horacio Cabral, Triantafyllos Stylianopoulos

**Affiliations:** ^1^ Cancer Biophysics Laboratory Department of Mechanical and Manufacturing Engineering University of Cyprus Nicosia 1678 Cyprus; ^2^ Department of Bioengineering Graduate School of Engineering The University of Tokyo Bunkyo Tokyo 113‐8656 Japan; ^3^ Centre for Stem Cell Research (A unit of inStem Bengaluru) Christian Medical College Campus Bagayam Vellore 560065 India; ^4^ Department of Hepatobiliary‐Pancreatic Surgery National Cancer Center Hospital East Kashiwa Chiba 277‐8577 Japan; ^5^ Department of Life Sciences Program in Biological Sciences European University Cyprus Nicosia 2404 Cyprus; ^6^ The Center for the Study of Haematological and other Malignancies Nicosia 2032 Cyprus; ^7^ Karaiskakio Foundation Nicosia 2032 Cyprus; ^8^ Cyprus Cancer Research Institute Nicosia 2032 Cyprus; ^9^ Exploratory Oncology Research and Clinical Trial Center National Cancer Center Kashiwa Chiba 277‐8577 Japan; ^10^ Medical School University of Cyprus Nicosia 1678 Cyprus; ^11^ Bank of Cyprus Oncology Centre Nicosia 2012 Cyprus; ^12^ Innovation Center of NanoMedicine Kawasaki Institute of Industrial Promotion Kawasaki Kanagawa 210‐0821 Japan; ^13^ Institute for Future Initiatives The University of Tokyo Bunkyo Tokyo 113‐0033 Japan

**Keywords:** immune checkpoint inhibition, nanomedicine, stroma normalization, tumor microenvironment, vascular normalization

## Abstract

Nano‐immunotherapy regimens have high potential to improve patient outcomes, as already demonstrated in advanced triple negative breast cancer with nanoparticle albumin‐bound paclitaxel and the immune checkpoint blocker (ICB) atezolizumab. This regimen, however, does not lead to cures with median survival lasting less than two years. Thus, understanding the mechanisms of resistance to and development of strategies to enhance nano‐immunotherapy in breast cancer are urgently needed. Here, in human tissue it is shown that blood vessels in breast cancer lung metastases are compressed leading to hypoxia. This pathophysiology exists in murine spontaneous models of triple negative breast cancer lung metastases, along with low levels of perfusion. Because this pathophysiology is consistent with elevated levels of solid stress, the mechanotherapeutic tranilast, which decompressed lung metastasis vessels, is administered to mice bearing metastases, thereby restoring perfusion and alleviating hypoxia. As a result, the nanomedicine Doxil causes cytotoxic effects into metastases more efficiently, stimulating anti‐tumor immunity. Indeed, when combining tranilast with Doxil and ICBs, synergistic effects on efficacy, with all mice cured in one of the two ICB‐insensitive tumor models investigated is resulted. These results suggest that strategies to treat breast cancer with nano‐immunotherapy should also include a mechanotherapeutic to decompress vessels.

## Graphical abstract

Combinatorial treatment of a mechanotherapeutic and nanomedicine normalizes metastasis microenvironment by decreasing fibrosis and hypoxia and skewing TAMs polarization from immunosuppressive M2‐like TAMs to immunostimulatory M1‐like TAMs. Improved perfusion and oxygenation leads to improved efficacy of immune checkpoint blockers and cure.

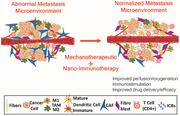

## 1 Introduction

Immune checkpoint blockade (ICB) has been approved as a monotherapy or in combination with other therapies for several types of solid tumors, including metastatic triple negative breast cancer.^[^ ^1^, ^2^ ^]^ The use of antibodies against programmed cell death protein 1 and its ligand (anti‐PD‐1/PD‐L1) or cytotoxic T lymphocyte‐associated protein 4 (anti‐CTLA‐4) is changing the standard of care in cancer treatment and currently there are numerous ongoing clinical trials employing ICB, which are expected to extend its clinical application to more tumor types.^[^ ^3^, ^4^ ^]^ However, it is estimated that less than 15% of cancer patients benefit from ICB monotherapies,^[^ ^2^ ^]^ while immunotherapy has failed to lead to durable therapeutic outcomes in triple negative breast cancer. Therefore, there is a clinical need to optimize ICB use in these tumors.

It has been well recognized that the hypo‐perfused and hypoxic microenvironment of solid tumors imposes major barriers to the efficacy of immunotherapies.^[^ ^3^, ^5^, ^6^, ^7^, ^8^ ^]^ Hypo‐perfusion, owing to the compromised functionality of the tumor vasculature, inhibits the systemic delivery and uniform intratumoral distribution of ICBs.^[^ ^3^, ^6^ ^]^ In addition, hypo‐perfusion causes hypoxia, which in turn induces immunosuppression through several mechanisms.^[^ ^9^, ^10^, ^11^, ^12^, ^13^ ^]^ Specifically, hypoxia promotes checkpoint expression,^[^ ^14^, ^15^ ^]^ and reprograms tumor‐associated macrophages (TAMs) from an immunosupportive M1 towards an immunosuppressive M2‐phenotype and hinders dendritic cell maturation that is required for tumor‐associated antigen presentation and activation of effector immune cells.^[^ ^9^, ^10^, ^16^ ^]^ Additionally, hypoxia affects the function of cytotoxic T cells reducing their killing potential,^[^ ^17^, ^18^ ^]^ whereas the dense/stiff tumor extracellular matrix (ECM), consisting mainly of collagen and hyaluronan, acts as a physical barrier for the infiltration of T cells into the tumor.^[^ ^7^, ^19^, ^20^, ^21^ ^]^ Furthermore, the tumor ECM along with the fibroblasts of the tumor contribute to the development of intratumoral mechanical forces that can cause the compression of tumor blood vessels and thus, hypo‐perfusion.^[^ ^22^, ^23^, ^24^, ^25^ ^]^

Higher perfusion levels have been related to improved efficacy of ICB treatment.^[^ ^26^ ^]^ Normalization of tumor stroma with the use of mechanotherapeutics (e.g., losartan, tranilast, pirfenidone, dexamethasone) has the potential to repair abnormalities of the tumor vasculature and thus, restores tumor perfusion and oxygenation, and improves anti‐tumor immunity.^[^ ^27^, ^28^, ^29^, ^30^, ^31^, ^32^, ^33^ ^]^ Recent studies in animal tumor models of breast cancer have shown that stroma normalization methods employing valsartan‐loaded nanoparticles, blocking CXCR4 or combining tranilast‐Doxil improve the efficacy of ICBs.^[^ ^34^, ^35^, ^36^, ^37^ ^]^ In particular, we have shown that the antihistamine and anti‐fibrotic drug tranilast, a known TGF‐*β* inhibitor, combined with the approved pegylated liposomal doxorubicin, Doxil, can optimize normalization of the primary breast tumor micro‐environment, improving perfusion. Furthermore, we found that ICB can significantly delay primary breast tumor growth rate when combined with tranilast‐Doxil normalization.^[^ ^36^ ^]^ Interestingly, these effects were not observed when tranilast was combined with the conventional doxorubicin, presumably owing to the superior pharmacokinetic properties of Doxil. However, there is a lack of data about the effect of stroma normalization on the vascular functionality of the metastatic microenvironment. Treating of the metastatic tumors is crucial for successful therapy and is associated with major barriers to the efficacy of nanomedicine and immunotherapy.

Metastatic cancer cells can bring their own stromal components, including activated fibroblasts, from the primary site to the lungs,^[^ ^38^ ^]^ thus creating a fibrotic, desmoplastic microenvironment similar to the primary tumor from which they arose.^[^ ^39^, ^40^ ^]^ Therefore, blood vessel abnormalities are expected to be found in metastatic vessels, limiting perfusion in metastatic tumors, but which abnormalities and how they vary with host organ are unclear.^[^ ^41^, ^42^, ^43^ ^]^ To this end, using metastasis data from patients and mice, we confirm that vessel compression exists in breast cancer lung metastases and demonstrate in murine models that stroma normalization using tranilast leads to improved perfusion as well as increased anti‐tumor activity of Doxil‐nanomedicine. Additionally, tranilast‐Doxil combination further improves perfusion and increases immunogenic cell death in lung metastases, enhances the immune metastatic microenvironment and improves the efficacy of ICBs in metastatic 4T1 and E0771 triple negative breast cancer models. Importantly, all mice bearing E0771 tumors that received combined treatment with tranilast and nano‐immunotherapy (Doxil + ICB) survived and were cured as indicated by the failure of tumor growth after cancer cell re‐implantation and the complete lack of lung metastatic lesions.

## 2 Results

### 2.1 Vessel Compression Occurs in Breast Cancer Lung Metastases Leading to Hypoxia

**Figure 1:**
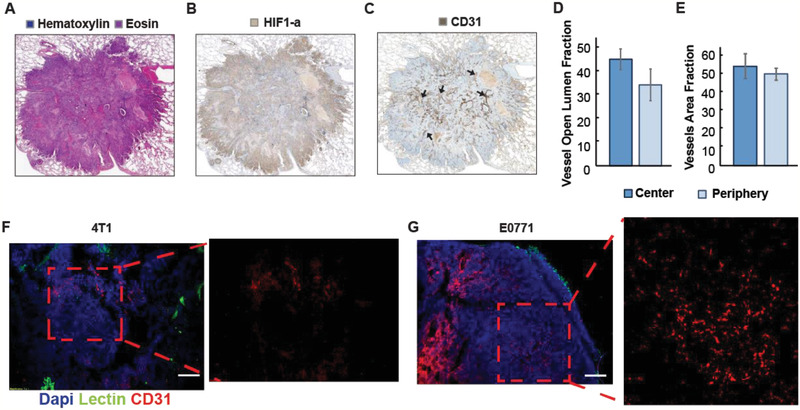
Vessel compression occurs in human and murine breast tumor lung metastases. A) Histology sample of lung metastasis from human bearing advanced breast cancer as indicated by H&E staining. B) HIF1‐a immunostaining (brown) indicating hypoxic regions of lung metastasis. C) Vasculature of metastatic lung nodules as shown by CD31 immunostaining (brown). Arrows demonstrate vessel compression. D) Quantification of open lumen fraction in the center and periphery of human metastases as indicative of vessel compression. E) Quantification of CD31 endothelial cell clusters with and without lumen. F,G) Representative fluorescence images of 4T1 and E0771 metastatic nodules immunostained for biotinylated tomato lectin (green), CD31 (red) and DAPI (blue).

We first evaluated lung metastases from eight breast cancer patients (**Figure** **1**A–E). The metastatic tumors had excessive levels of hyaluronan and collagen, were hypoxic and their vessels were irregular (Figure 1B,C and Figure S1, Supporting Information). We quantified the compression as the fraction of the vessels with open lumen (Figure 1D) and found that the fraction of vessels with open lumen was similar to previous studies in mouse primary breast tumors.^[^ ^27^ ^]^ Also, the amount of compression did not vary based on proximity to the edge of the lesion. The microvascular density also did not vary with location (Figure 1E).

**Figure 2:**
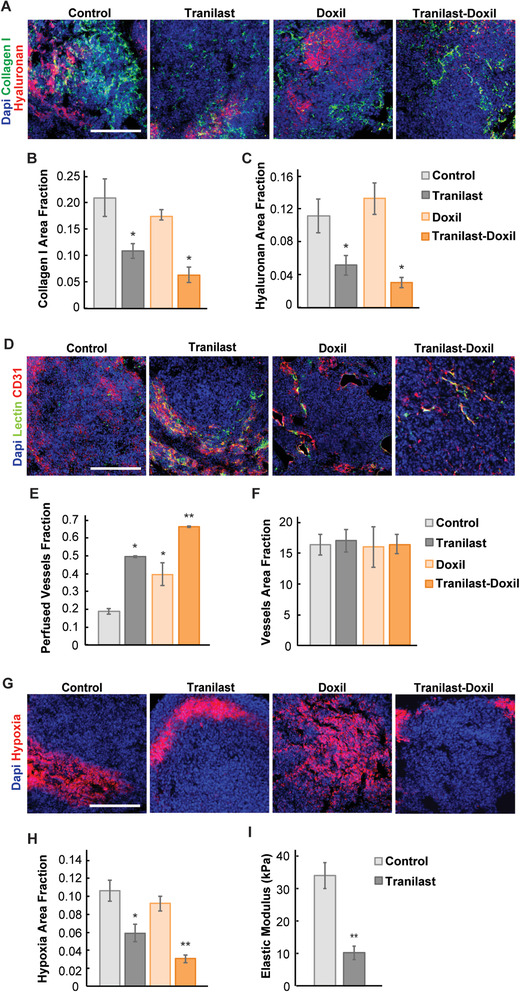
Tranilast and Doxil treatment normalizes the microenvironment of metastases by reducing collagen and hyaluronan content, and by increasing perfusion and oxygenation. A) Representative fluorescence images of collagen I (green), hyaluronan (red) immunostaining and DAPI nuclear staining of 4T1 metastatic nodules treated as indicated. B) Quantification of collagen I and hyaluronan (C) area fraction in the interstitial microenvironment of lung nodules. D) Representative fluorescence images of 4T1 metastatic nodules immunostained for biotinylated tomato lectin (green), CD31 (red), and DAPI (blue) after various treatments. E) Quantification of vascular perfusion indicated by the co‐expression of CD31 endothelial marker and lectin (yellow). F) Vessel area fraction as defined by CD31 (red) positive staining. G) Representative fluorescence images of E0771 metastatic lung nodules immunostained with pimonidazole (hypoxia, red), and DAPI (blue) nuclear staining. H) Quantification of hypoxic fraction following pimonidazole (60 mg kg^–1^) injection and staining. I) Elastic modulus measurements of E0771 lung metastases for the control and tranilast treated group. Statistical analyses were performed by comparing the treated groups with the control * and the tranilast‐Doxil groups with all other treatment groups **, *p* < 0.05, (*n* = 8–10). Scale bar: 200 µm.

Subsequently, we sought to confirm that metastatic murine breast cancer recapitulates the pathophysiology we observed in patients. We employed two spontaneous triple negative breast cancer metastasis models of syngeneic 4T1 and E0771 tumors by inoculating cancer cells in the mammary fat pad and then removing the tumor after allowing enough time for lung metastases to develop. Vessel compression was identified by immunofluorescence staining of lung sections with biotinylated lectin and the endothelial marker CD31. As lectin was administered intracardially prior to tumor removal, it bound to the endothelial cells of the functional (perfused) vessels, whereas staining of tumor sections with the CD31 marker stained both functional and collapsed vessels owing to compression (Figure 1F,G). Therefore, perfused vessels can be identified by positive staining in both lectin and CD31. We found that lung metastases have abundant non‐functional, compressed vessels, which should limit drug delivery and oxygen supply. It was also confirmed that lung metastasis had sufficient levels of collagen and hyaluronan (**Figure** **2**A,B; Figure S2, Supporting Information), which are major contributors to vessel compression and hypo‐perfusion.^[^ ^23^, ^25^, ^27^, ^30^ ^]^ Therefore, vessel compression occurs in both human and murine metastatic breast tumors.

### 2.2 The Mechanotherapeutic Tranilast and Doxil Nanomedicine Normalize Metastasis Vasculature

We next investigated if the mechanotherapeutic drug tranilast can improve vessel functionality by reducing ECM levels similar to its effects in primary murine breast tumors.^[^ ^29^ ^]^ Apart from tranilast, Doxil can act as a metronomic chemotherapy and normalizes the tumor vessels like an antiangiogenic agent.^[^ ^3^, ^36^, ^44^ ^]^ Tranilast reduced collagen and hyaluronan content and perfused vessels, as indicated by lectin and CD31 positive staining, demonstrated that perfused vessel fraction increased significantly in the tranilast‐treated tumors without affecting the total number of vessels (Figure 2A–F; Figure S2, Supporting Information). As a consequence of improved perfusion, oxygen delivery was enhanced and pimonidazole staining detected a significant decrease in the hypoxic fraction (Figure 2G,H). We also found that the reduction of collagen and hyaluronan resulted in the softening of the lung metastases, reducing significantly their elastic modulus (Figure 2I). As far as Doxil treatment is concerned, we found that Doxil improves perfusion only in the 4T1 and not the E0771 metastases but the combination of tranilast‐Doxil optimizes the normalization effects of tranilast, improving further ECM reduction, perfusion and oxygenation (Figure 2; Figure S2, Supporting Information). Furthermore, tranilast‐Doxil treatment increased the pericyte coverage of the tumor blood vessels, which is a measure of normalization of the tumor vessel wall (Figure S3, Supporting Information). Therefore, tranilast repairs vessel abnormalities in the metastatic vasculature and its combination with Doxil optimizes the normalization effects.

### 2.3 Normalization Increases Spatial Distribution of Doxil‐Induced Cell Killing, Potentiates Doxil Against Lung Metastasis, and Enhances Immune Microenvironment

**Figure 3:**
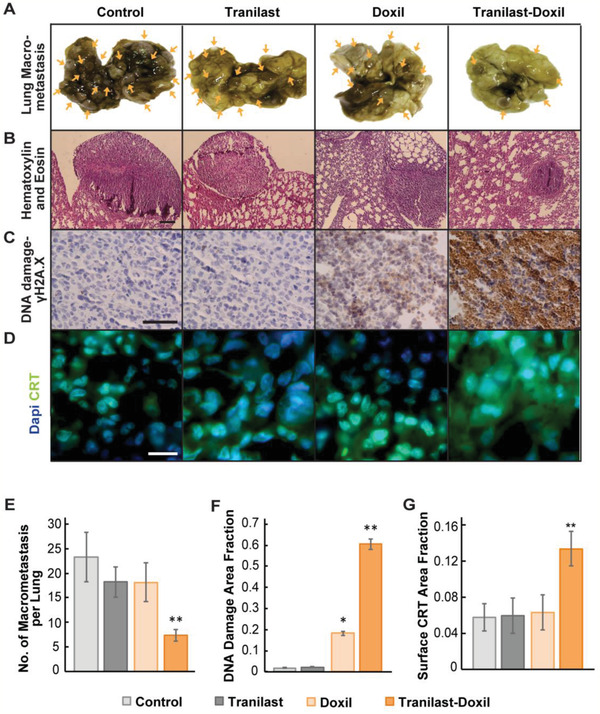
Combinatorial tranilast‐Doxil treatment reduces lung metastasis significantly. A) Lung photographs of 4T1 murine breast tumors treated as indicated. Yellow arrows indicate the macro‐metastatic nodules on the lungs. B) Representative H&E histopathological images of lungs bearing 4T1 metastases. C) Representative images of lung tumors after *γ*H2A.X immunostaining (brown) indicating the DNA double strand breaks‐induced cell death and Hematoxylin nuclear staining (blue). Scale bar: 200 µm. D) Representative lung macrometastasis images of calreticulin immunostaining (green) and dapi (blue). Scale bar: 10 µm. E) Quantification of 4T1 nodules counted in both sides of lung (front and back) under a stereoscopic microscope after treated as indicated. F) Quantification of *γ*H2A.X area fraction, representing DNA damage in 4T1 lung macrometastases. G) Immunogenic cell death of 4T1 metastasis as indicated by the quantification of surface CRT. Statistical analyses were performed by comparing the treated groups with the control * and the tranilast‐Doxil group with all other treatment groups **, *p* < 0.05, (*n* = 8–10).

Subsequently, we examined the ability of Doxil to reduce metastasis with and without tranilast in mice bearing 4T1 and E0771 breast tumors. Macroscopic counting of lung metastatic nodules, as well as, histological analysis of tissue morphology by Hematoxylin and Eosin (H&E) staining showed reduced metastasis (**Figure** **3**A,B,E; Figure S4, Supporting Information). Remarkably, only the tranilast‐Doxil treatment could effectively inhibit lung macrometastases formation in both tumor models compared to all other treatment groups, both in terms of the number and size of metastases. An explanation for the reduced number of metastases is that the improved vessel functionality induced by normalization results in improved efficacy of the nanomedicine. Therefore, we performed immunohistochemical analysis of DNA damage using antibodies directed against the *y*H2A.X, a DNA double‐strand break marker (Figure 3C). DNA damage was improved more than threefold when Doxil was combined with tranilast compared to the other treatments (Figure 3C,F) and it was uniformly distributed throughout the tumor. Taking into account that doxorubicin, that is, the cytotoxic payload of Doxil, induces immunogenic cell death, we sought to examine the immunogenic effects of Doxil on metastasis via quantification of the surface calreticulin (CRT) on dying cancer cells ^[^ ^45^ ^]^ (Figure 3D). Consistent with the DNA damage‐induced cell death results, only the tranilast‐Doxil combined treatment could efficiently stimulate immunogenic cell death (Figure 3G). We also noticed that these effects of tranilast‐Doxil on reducing metastasis, increasing cancer cell killing, and inducing immunogenic cell death were not observed when tranilast was combined with the free drug, doxorubicin (Figure S5, Supporting Information).

**Figure 4:**
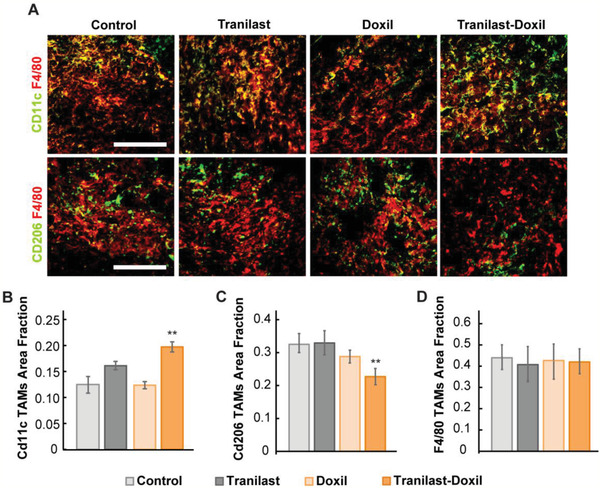
Tranilast‐Doxil combinatorial treatment induces immunostimulation in lung macrometastases of E0771 breast tumor model, by directing macrophages polarization towards M1 phenotype. A) Representative images of E0771 lungs bearing macrometastatic nodules immunostained for either the M1‐like tumor associated macrophage (TAM) maker CD11c (green) or the M2‐like TAM marker CD206 (green), and F4/80, which is a pan‐macrophage marker (red). CD11c and CD206 quantification was performed by calculating the area of CD11c^+^ and CD206^+^ to the overlapping F4/80^+^ signal (yellow color) per F4/80^+^ signal. Quantification of total macrophage population (F4/80 signal, red) was performed following normalization to DAPI positive area fraction. Quantification of anti‐tumoral M1‐like TAMs (B), pro‐tumoral M2‐like TAMs (C) and total TAMs fraction (D) in the metastatic nodules among the various treatment groups. Statistical analyses were performed by comparing the treated groups with the control * and the tranilast‐Doxil group with all other treatment groups **, *p* < 0.05, (*n* = 8–10). Scale bar: 200 µm.

We also hypothesized that the increased perfusion and oxygenation of the lung metastases caused by the tranilast‐Doxil normalization treatment can improve immunostimulation by increasing the population of M1 macrophages. Analysis of immunofluorescence staining images of tumor sections for M1‐like, M2‐like, and total TAMs showed an increase in the area fraction of M1‐like TAMs and a decrease in the levels of pro‐tumor M2‐like TAMs (**Figure** **4**) confirming the hypothesis.

Taken all together, these findings indicate that normalization of the tumor stroma can facilitate nanomedicine efficacy to lung metastases, increasing immunogenic cell death. Furthermore, increased perfusion and oxygenation can stimulate M1‐like TAMs.

### 2.4 Normalizing the Microenvironment Overcomes Resistance to Nano‐Immunotherapy

**Figure 5:**
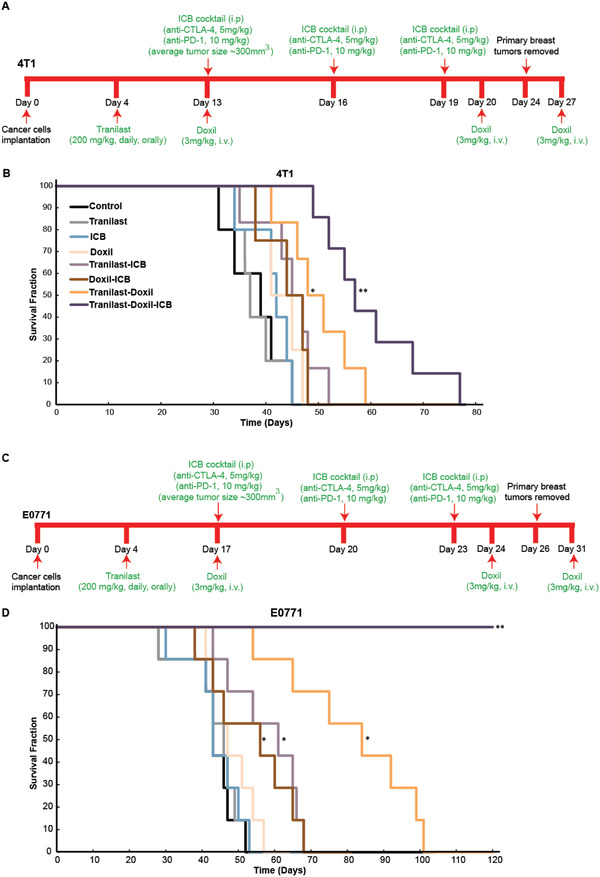
Tranilast‐induced normalization increases the efficacy of nano‐immunotherapy. Kaplan–Meier survival curves for 4T1 (A, B) and E0771 (C, D) tumor models treated as indicated (arrows). Statistical analyses were performed by comparing the treated groups with the control * and the tranilast‐Doxil groups with all other treatment groups **, *p* < 0.05 (*n* = 7–9).

Subsequently, we investigated the antitumor effects of normalization combined with ICB therapy. For this study, tranilast was combined with Doxil and a mixture of blocking antibodies against the immune checkpoints cytotoxic T lymphocyte‐associated protein 4 (anti‐CTLA‐4) and programmed cell death 1 (anti‐PD‐1) to treat mice bearing 4T1 and E0771 tumors. We first employed an overall survival study according to the treatment protocols presented in **Figure** **5**A,C. Combination of tranilast with Doxil and ICB resulted in a significant increase in the overall survival (time to death) of the mice compared to all other treatments for both cancer cell lines (Figure 5B,D). Particularly in the E0771 tumors, the triple therapy had a dramatic effect on overall survival since all mice of this group survived.

**Figure 6:**
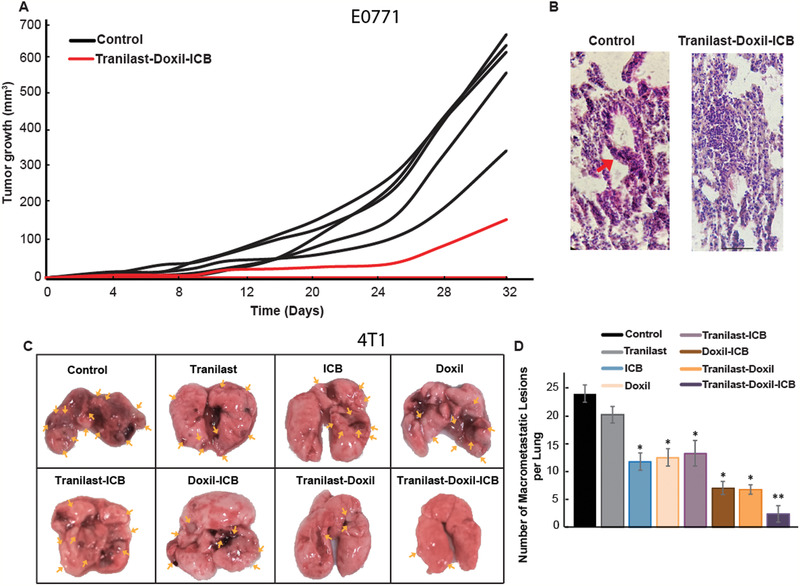
Mice treated with tranilast and nano‐immunotherapy gained immunological memory to cancer‐associated antigens. Given that 120 days from the initiation of the overall survival study, all mice bearing E0771 tumors (*n* = 9) had survived following treatment with tranilat‐Doxil and the ICB cocktail. E0771 cells were re‐implanted in a different mammary fat pad and a group of mice of the same age was employed as control (*n* = 5). A) Tumor growth curves of the control and tranilast‐Doxil‐ICB treated mice. All tumors grew in the control mice, whereas in the treated group a tumor grew only in one of the nine mice. B) Representative H&E images of lung tissue indicates formation of micrometasasis in the control group (left), whereas almost all treated mice had no signs of lung metastasis (right). C) Tranilast and nano‐immunotherapy significantly decreases lung macrometastases. Representative lung images of 4T1 breast tumors treated as indicated. Yellow arrows depict the macrometastatic nodules on the lungs. D) Quantification of nodules counted in both sides of lung (front and back) under a stereoscopic microscope after treated as indicated.

At this point, E0771 cells were implanted again in a different mammary fat pad of the mice that had received the triple therapy and another group of mice of the same age were employed as a control to investigate if a primary tumor would be developed in the treated mice. Remarkably, even though a tumor grew in all control mice, only one grew in the treated mice (in 1 out of 9 mice) within the time period of the experiment (**Figure** **6**A). When the tumors of the control group reached an average volume of approximately 500 mm^3^, we terminated the experiment and collected the lungs of both groups for H&E staining (Figure 6B). Even though metastatic lesions formed in one of the control mice, all treated mice exhibited no evidence of metastasis. We concluded that the combination of tranilast with Doxil and ICB treatment provides evidence of immunological memory of the mice to E0771 cancer cells.

### 2.5 Tranilast Combined with Nano‐Immunotherapy Eliminates Lung Metastases and Promotes Immunostimulation

To investigate the mechanism of the improved therapeutic outcomes of the triple therapy, we repeated the experiment in 4T1 tumors combining tranilast, Doxil, and the ICB cocktail but this time we removed the lungs of all groups 10 days after the resection of the primary tumors. Our analysis involved macroscopic counting of the metastatic lesions, assessment of T cell infiltration via immunohistochemistry experiments and flow cytometry analysis of T cells and macrophages. The tumors treated with tranilast and nano‐immunotherapy had a significant decrease in the number of macrometastases compared to all other groups (Figure 6C,D). It has also been proposed that ICB may decrease the levels of vascular endothelial growth factor (VEGF) and thereby normalize the tumor vessel wall indirectly.^[^ ^10^, ^37^, ^46^ ^]^ We evaluated the effect of immunotherapy on the normalization of the vessel wall by the fraction of pericyte coverage on blood vessels. Interestingly, we found that immunotherapy alone caused a slight increase in pericytes on vessels wall as indicated by the CD31 and NG2 overlapping staining, while its combination with the normalization agent tranilast or the tranilast‐Doxil combined treatment significantly enhanced blood vessel integrity compared to the other treatment groups (Figure S3, Supporting Information).

**Figure 7:**
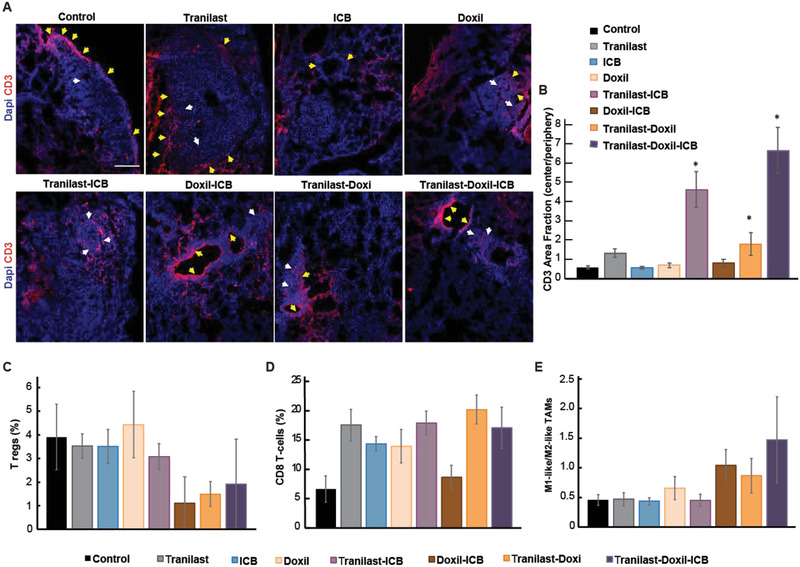
Tranilast combined with nano‐immunotherapy promotes immunostimulation. A) Representative fluorescence images of 4T1 lung metastasis slices immunostained for the CD3 immune T cell marker (red, CD4^+^ and CD8^+^ T cells) and DAPI (blue, cell nuclei). Yellow arrows indicate accumulation of T cells at the periphery while white arrows demonstrate immune cell infiltration within the metastatic lesion. B) Efficacy of T cell infiltration was quantified by the ratio of central to peripheral CD3 positive staining normalized to DAPI staining, (*n* = 8–10). Flow cytometry analysis of C) CD3^+^CD4^+^CD127^lo^CD25^hi^Foxp3^+^ Tregs among total T cells, D) cytotoxic CD8^+^ T cells among total T cells and E) ratio of M1‐like (CD45^+^ CD11b^+^ Gr1^−^ F4/80^+^ CD11c^+^ CD206^−^) to M2‐like (CD45^+^ CD11b^+^ Gr1^−^ F4/80^+^ CD11c^−^ CD206^+^) over total TAMs of metastatic lung lesions of 4T1 breast tumors, (*n* = 5). Statistical analyses were performed by comparing the treated groups with the control *, *p *< 0.05, and the tranilast‐Doxil‐ICB group with all other treatment groups **, *p *< 0.05. Scale bar: 200 µm.

The tranilast‐induced stroma normalization effect was also evident by the increased CD3^+^ T cell infiltration to the metastatic lesions. Specifically, the dense ECM poses a physical barrier to the penetration of immune cells within the tumor microenvironment. Here, we observed that combination of tranilast or tranilast‐Doxil with the ICB cocktail increased the number of CD3^+^ T cells at the center of the tumor, enhancing their migration through the tumor microenvironment (**Figure** **7**A,B and Figure S6, Supporting Information). Therefore, high ECM density might contribute to an immune‐excluded tumor phenotype in breast cancer lung metastases, characterized by the inability of immune cells to penetrate deep into the tumor and approach cancer cells.^[^ ^20^ ^]^

Further examination of T cell population infiltrated in the metastatic lesions via flow cytometry analysis revealed a decrease trend in the percentage of the immunosuppressive regulatory T cells (Tregs), and an increase trend in the ratio of M1‐like to M2‐like macrophages for the tranilast‐Doxil, Doxil‐ICB, and tranilast‐Doxil‐ICB groups (Figure 7C,E). No difference was observed in the population of cytotoxic CD8^+^ T cells among the treatment groups (Figure 7D).

Collectively our data indicate that immunotherapy is more efficient when administered in combination with a normalization agent, whereas the combination with a cytotoxic agent such as Doxil is required for improved therapeutic effects.

## 3 Discussion

Normalization of the tumor stroma is an emerging strategy that has already succeeded as “potentially curative” in a phase II trial in locally advanced (i.e., non‐metastatic) desmoplastic pancreatic cancer.^[^ ^47^ ^]^ Use of mechanotherapeutics to induce stroma normalization involves targeting of cancer‐associated fibroblasts and extracellular collagen and hyaluronan, which in turn alleviates intratumoral forces, decompresses tumor vessels, and improves perfusion as well as the systemic delivery of cytotoxic agents. Most pertinent studies, however, have focused mainly on primary tumors. Here, using metastasis data from patients and mice, we demonstrated that vessel compression occurs in breast cancer lung metastases causing hypo‐perfusion and hypoxia. Furthermore, based on our murine lung metastasis data, we made the following advances: i) identified high ECM levels and vessel compression as the dominant mechanism of hypo‐perfusion in desmoplastic breast cancer lung metastases, which in turn causes resistance to nanomedicine, ii) demonstrated that stroma normalization overcomes this resistance, increasing efficacy of Doxil‐nanomedicine to lung metastases, iii) showed that combination of stroma normalization agents and cytotoxic nanomedicine seems to enhance the metastatic immune microenvironment, and iv) found that combination of stroma normalization with nano‐immunotherapy improves drastically the therapeutic outcome and might lead to immunological memory to cancer‐associated antigens. The latter is of particular importance given that a phase II clinical study for the combined use of normalization using the mechanotherapeutic losartan with ICB (Nivolumab), chemotherapy (FOLFIRINOX) and radiation therapy has been already initiated (ClinicalTrials.gov Identifier: NCT03563248). Losartan belongs to a class of anti‐hypertensive therapies (i.e., angiotensin receptor blockers) repurposed for cancer. Patients might be normo‐ or hypo‐tensive and thus, might not be able to tolerate anti‐hypertensive therapies. To overcome this problem, this study uses the antihistamine and anti‐fibrotic drug tranilast, which also interferes with TGF‐*β* signaling.^[^ ^29^ ^]^ Importantly, tranilast is approved in Japan and South Korea and thus, it could be safely and rapidly tested in hypo‐ and normotensive patients who cannot receive losartan.

Our results also have implications for ongoing clinical trials of cancer nanomedicines, as they describe how TGF‐*β* inhibition can normalize metastases and in turn improve nanocarrier efficacy to metastatic breast cancer and stimulate anti‐tumor immune responses. In the current study, we employed two metastatic triple negative syngeneic models, 4T1 and E0771 and human data to demonstrate that vessel compression exists in breast cancer lung metastases in patients and mice, yet it can be reversed with combined stroma normalization and nanomedicine. Indeed, tranilast in combination with Doxil caused a pronounced reduction in the ECM components and an increase in the diameter of intratumoral blood vessels resulting in a significant increase in perfusion in the metastatic lesions. Improved perfusion in metastatic nodules resulted in increased nanomedicine efficacy, demonstrated by Doxil‐induced DNA damage, immunogenic cell death, and reduction of in metastatic burden. Increased nanomedicine efficacy could have been also enabled by the decrease in ECM components, allowing the nanoparticles to penetrate into the tumor.^[^ ^48^ ^]^ We further demonstrated that the combination of tranilast, Doxil, and ICB drastically enhances the efficacy of both Doxil and ICB and can lead to cure. Additionally, according to Figures 2 and 7A,B, a decrease in extracellular matrix levels and alleviation of hypoxia results in an increase in T cell infiltration. Overall, these results reveal a new mechanism of treatment resistance of breast cancer lung metastases, and that the combination of stroma normalization and nanomedicine overcomes this resistance.

## 4 Experimental Section

A detailed description of all materials and methods employed in the study are presented in the Supporting Information. Here, a summary is provided.

##### Animal Tumor Models and Treatment Protocols

4T1 and E0771 syngeneic tumor models were generated by orthotopic implantation of either 5 × 10^4^ 4T1 or 5 × 10^4^ E0771 mouse breast cancer cells in 40 µL of serum‐free medium into the mammary fat pad of 6‐week old BALB/c and C57BL/6 female mice, respectively. A neoadjuvant treatment was modeled and animals were treated with saline (Control), tranilast (200 mg kg^−1^, orally, daily, 4 days post implantation), Doxil, (3 mg kg^−1^, intravenously weekly and for 3 weeks after tumors reached an average size of 300 mm^3^) or tranilast‐Doxil.^[^ ^27^, ^29^ ^]^ Primary tumors of all groups were surgically resected at the time when they reached a minimum size of 1200 mm^3^, which is considered sufficient time for metastatic tumors to be formed.^[^ ^34^, ^35^ ^]^ Subsequently, the tissue was sutured for the study of the metastatic tumors. For the overall survival studies, the ending point was the time to mouse death. For the study of the alterations of the metastatic microenvironment, the lungs were collected for all groups at the same time, 10 days following the resection of the primary tumors.

Tumor size was measured every 2–3 days and tumor volume was calculated from the volume of ellipsoid, measuring the two dimensions (x,y) and assuming the third dimension to be equal to $\sqrt{xy}$. Prior to lungs excision, animals were anesthetized via Avertin (200 mg kg^−1^, intraperitoneal) and intracardially slowly injected with biotinylated lycopersicon esculentum lectin (4 mg kg^−1^) and/or pimonidazole HCl (60 mg kg^−1^, intraperitoneal) that were used for perfusion and hypoxia measurements.^[^ ^27^, ^29^, ^31^ ^]^

Immunotherapy was administrated as a cocktail of 10 mg kg^−1^ anti‐PD‐1 (CD279, clone RMP1‐14, BioXCell) and 5 mg kg^−1^ anti‐CTLA‐4 (CD152, clone 9D9) following dilution in the recommended in vivo pure pH 7.0 Dilution Buffer (BioXCell).^[^ ^34^, ^35^, ^36^ ^]^ The immunotherapy cocktail was administered i.p. when tumors reached an average size of 300 mm^3^ every three days for three doses. For the immunotherapy studies, animals were also treated with a non‐targeting isotype control antibody (BioXCell). All in vivo experiments were conducted in accordance with the animal welfare regulations and guidelines of the European Union (European Directive 2010/63/EE and Cyprus Legislation for the protection and welfare of animals, Laws 1994–2013) under a license acquired and approved (No CY/EXP/PR.L2/2018, CY/EXP/PR.L14/2019, CY/EXP/PR.L15/2019) by the Cyprus Veterinary Services committee, the Cyprus national authority for monitoring the welfare of animals in research.

##### Biomechanical Analysis

Characterization of the mechanical properties and calculation of the elastic modulus of the metastatic lesions was determined using an unconfined compression experimental protocol as previously described.^[^ ^24^, ^25^ ^]^

##### Immunohistochemical Assessment of Metastasis Microenvironment

For fluorescent immunohistochemistry and vessel perfusion histology experiments, lungs were excised, fixed with 4% PFA and embedded in optimal temperature compound (OCT) to produce transverse 40 µm‐thick cryosections. ECM content was studied using immunostaining against collagen I and hyaluronan, while vascular perfusion was examined following staining against the CD31 endothelial marker and biotinylated tomato lectin. Hypoxic regions within primary tumor and metastatic nodules were detected using the mouse anti‐pimonidazole RED 549 conjugate antibody. Pericyte coverage of blood vessels was determined by the co‐localization of NG2 (AB5320, Millipore 1:200) and CD31 positive staining normalized to total CD31 area fraction. Macrophage status of E0771 TME was determined following immunostaining with hamster anti‐CD11c (HL3, BD Pharmingen 1:100), rabbit anti‐CD206 (ab64693, Abcam 1:100) and rat anti‐F4/80 (A3‐1, BIO‐RAD 1:50) antibodies to detect M1‐like TAMs, M2‐like TAMs, and total TAM population, respectively. CD11c and CD206 quantification was performed by calculating the area of CD11c^+^ and CD206^+^ to the overlapping F4/80^+^ signal (yellow color, Figure 4) per F4/80^+^ signal. Quantification of total F4/80 signal was performed following normalization to DAPI positive area fraction. T cell infiltration within the TME of lung metastatic lesions was determined as the ratio of central CD3 (rat anti‐CD3 (17A2, BioLegend 1:100) positive staining to peripheral following normalization to DAPI.

##### Flow Cytometry

On day 25 of treatment, 4T1 macrometastatic lesions were harvested in 1x PBS, minced to fine fragments and incubated with Accumax (Millipore) for 1 h at room temperature on an end‐over‐end shaker. Enzymatic digestion was ceased by the addition of RPMI media containing 10% FBS and 1% antibiotic/antimycotic solution. The resulting tissue homogenates were filtered through 70 µm cell strainers and single cells suspensions were collected and counted. Cell suspensions were then incubated with fixable viability dye (Invitrogen) for gating of viable cells. Non‐specific antibody binding was blocked following incubation with the rat anti‐mouse CD16/CD32 mAb (BD Bioscience) for 10 min at room temperature. 1 × 10^6^ cells per sample were labeled with the various fluorochrome conjugated antibodies, washed and resuspended in 1%BSA, 1xPBS buffer. The anti‐mouse antibodies used in the experiment are the following; CD4‐AF700 (GK1.5, BioLegend), CD127‐APC (A7R34, BioLegend), CD206‐PE‐Cy7 (C068C2, BioLegend), F4/80‐APC (BM8, BioLegend), IgG2a‐APC (BioLegend), IgG2b‐AF488 (BioLegend), IgG2a‐PE‐Cy7 (BioLegend), Cd11b‐eV450 (eBioscience), Gr1‐PE (RBG865, BioLegend), CD8a (53‐6.7, eBioscience), CD11c‐AF700 (HL3, BD Bioscience), Foxp3‐AF488 (MF23, BD Bioscience), CD45‐V500 (30‐F11, BD Bioscience), CD25‐PE‐Cy7 (PC61.5, BD Bioscience), CD3 (145‐2C11, BD Bioscience). Flow cytometry data were obtained using BD FACSAria III flow cytometer and analyzed using FlowJoX software. Data presented is representative of singlets, live cells.

##### Statistical Analysis

Data are presented as means with standard errors. Groups were compared using Student's *t*‐test to study statistical significance. Statistical analyses were performed by comparing the treated groups with the control * and the tranilast‐Doxil/ tranilast‐Doxil‐ICB group with all other treatment groups **. A *p*‐value of less than or equal to 0.05 was considered statistically significant.

## Author Contributions

F.M., M.P., and C.V. contributed equally to this work. The manuscript was written through contributions of all authors. All authors have given approval to the final version of the manuscript. F.M., M.P., C.V., R.S., S.T., N.G., To.S., P.D., C.P., and P.P. performed research and analyzed data. L.K., P.C., M.K., G.I., A.C. K.K., and H.C. supervised the study and analyzed data. J.D.M. and T.S. conceived and supervised the study and analyzed data.

## Conflict of Interest

The authors declare no conflict of interest.

## Supporting information

Supporting Information
