# Supplementary material for: Normalizing the Microenvironment Overcomes Vessel Compression and Resistance to Nano‐immunotherapy in Breast Cancer Lung Metastasis
Source: Adv Sci (Weinh). 2020 Dec 13;8(3):2001917. doi: 10.1002/advs.202001917 (PMC7856901; doi:10.1002/advs.202001917)
Supplement: Supplementary file 1 — Supporting Information [file ADVS-8-2001917-s001.pdf]

© 2020 Wiley-VCH GmbH

ADVANCED SCIENCE
Open Access

Supporting Information

for *Adv. Sci.,* DOI: 10.1002/advs.202001917

Normalizing the Microenvironment Overcomes Vessel Compression and Resistance to Nano-immunotherapy in Breast Cancer Lung Metastasis

Fotios Mpekris, Myrofora Panagi, Chrysovalantis Voutouri, John D. Martin, Rekha Samuel, Shinichiro Takahashi, Naoto Gotohda, Toshiyuki Suzuki, Panagiotis Papageorgis, Philippos Demetriou, Chryso Pierides, Laura Koumas, Paul Costeas, Motohiro Kojima, Genichiro Ishii, Anastasia Constantinidou, Kazunori Kataoka, Horacio Cabral, Triantafyllos Stylianopoulos

Supporting Information

Normalizing the Microenvironment Overcomes Vessel Compression and Resistance to Nano-immunotherapy in Breast Cancer Lung Metastasis

Fotios Mpekris, Myrofora Panagi, Chrysovalantis Voutouri, John D. Martin, Rekha Samuel, Shinichiro Takahashi, Naoto Gotohda, Toshiyuki Suzuki, Panagiotis Papageorgis, Philippos Demetriou, Chryso Pierides, Laura Koumas, Paul Costeas, Motohiro Kojima, Genichiro Ishii, Anastasia Constantinidou, Kazunori Kataoka, Horacio Cabral, Triantafyllos Stylianopoulos

Cell culture. 4T1 and E0771 mouse mammary carcinoma cell lines were purchased from ATCC and CH3 BioSystems, respectively. Both cell lines were maintained in Roswell Park Memorial Institute medium (RPMI, biosera) supplemented with 10% fetal bovine serum (FBS) and 1% antibiotics.

Drugs and reagents. Tranilast (Rizaben, Kissei Pharmaceutical, Japan) was dissolved in 1% $NaHCO_3$ (33.3 mg/ml) following incubation at 70°C for an hour, as previously described.[1-3] Doxil (Pegylated liposomal doxorubicin, Janssen Pharmaceuticals) was purchased as already made solution (2 mg/ml). The ICBs mouse monoclonal PD-1 (CD279, clone RMP1-14) and mouse monoclonal CTLA-4 (CD152, clone 9D9) were purchased from BioXCell.

Tumor volume. Planar dimensions (x, y) of tumor were monitored every 2-3 days using a digital caliper and tumor volume was estimated from the volume of an ellipsoid and assuming that the third dimension, z, is equal to $\sqrt{x\,y}$. Animal survival was quantified based on the time of death after initiation of treatment or time to reach maximum tumor burden of 1200 mm^3.[4] All *in vivo* experiments were conducted in accordance with the animal welfare regulations and guidelines of the Republic of Cyprus and the

European Union (European Directive 2010/63/EE and Cyprus Legislation for the protection and welfare of animals, Laws 1994-2013) under a license acquired and approved (No CY/EXP/PR.L2/2018, CY/EXP/PR.L14/2019, CY/EXP/PR.L15/2019) by the Cyprus Veterinary Services committee, the Cyprus national authority for monitoring animal research for all academic institutions.

Elastic modulus. Calculation of the elastic modulus were determined using an unconfined compression experimental protocol. Following excision of the primary tumor or the macroscopically metastatic nodules, specimens were loaded on a high precision mechanical testing system (Instron, 5944, Norwood, MA, USA) and compressed to a final strain of 30% with a strain rate of 0.1 mm/min. The metastatic nodules were tested as a whole owing to their small size. The elastic modulus was calculated from the slope of the stress-strain curve at the 25-30% strain range.[3, 5, 6]

Fluorescent immunohistochemistry. For immunohistochemistry (IHC) analysis of ECM constituents, 4T1 and E0771 lungs were removed, incubated with 4% paraformaldehyde (PFA, Sigma) in PBS for 40 min and washed twice for 10 min with 1xPBS. Fixed tissues were embedded in optimal cutting temperature compound (OCT) in cryomolds (Tissue-Tek) and allowed to freeze completely at -20°C. Transverse 40μm-thick of lung tissues were produced using the Tissue-Tek Cryo3 (SAKURA). Positively charged HistoBond® microscope slides (Marienfeld) were used to bound four lung sections with metastatic lesions per sample. Tissue sections were then incubated in blocking solution (10% FBS, 3% Donkey Serum, 1x PBS) for 2 hr and immunostained with the following primary antibodies; rabbit anti-collagen I (ab34710, Abcam 1:100), sheep anti-hyaluronic acid (ab53842, Abcam 1:100), hamster anti-CD11c (HL3, BD Pharmingen 1:100), rabbit anti-CD206 (ab64693, Abcam 1:100) and rat anti-F4/80 (A3-1, BIO-RAD 1:50), overnight at 4°C. Secondary antibodies against rabbit, sheep, rat or hamster conjugated to Alexa Fluor 488 and 647 (Invitrogen) were used at 1:400 dilution. All samples were incubated in secondary antibody solution including DAPI (Sigma, 1:100 of 1mg/ml stock) for 2 hr

at room temperature in the dark. Sections were mounted on microscope slides using the ProLong™ Gold Antifade Mountant (Invitrogen) and covered with glass coverslip.

Collagen I and Hyaluronan. Tissue cryosections of 4T1 and E0771 lungs were immunostained with anti-Collagen I (ab34710, Abcam 1:100) and anti-hyaluronic acid (ab53842, Abcam 1:100) and signal was detected with Alexa Fluor-647 anti-rabbit IgG (H+L) (A21244, Invitrogen 1:400) and Alexa Fluor-488 anti-sheep IgG (H+L) (A11015, Invitrogen 1:400) secondary antibodies, respectively.

Vascular perfusion. To assess functional vasculature, mice were anesthetized with i.p. injection of Avertin (200mg/kg) and slowly injected intracardially with 100 μl (4mg/kg) of biotinylated lycopersicon esculentum lectin (B-1175, Vector Labs) which was allowed to distribute throughout the body for 7 min.[4] Finally, mice were sacrificed via CO_2 inhalation and lungs were removed. Excised lungs were fixed in 4% PFA (Sigma) and processed as described above for IHC analysis. The number of blood vessels was measured from the positive staining of the endothelial marker CD31 (MEC13.3, BD Pharmingen 1:100) while the area fraction of perfused vessels was determined as the ratio of lectin and CD31 overlapping staining to CD31 positive staining. CD31 signal was detected with Alexa Fluor-647 Goat Anti-Rat IgG (H+L) (Invitrogen, A-21247, 1:400) secondary antibody and lectin staining with Streptavidin Alexa Fluor 488 conjugate (Invitrogen, S11223, 1:200 dilution).

Hypoxia studies. Mice bearing orthotopic E0771 breast tumors were injected with 60mg/kg of pimonidazole HCl at 10mg/ml 2 hr prior to lungs removal. Lungs were then excised, fixed in 4% PFA embedded in OCT and processed accordingly for IHC. Hypoxic regions were detected using the mouse anti-pimonidazole RED 549 conjugate antibody (HP7-100Kit, 1:100). Hypoxic area fraction across different treatment groups was normalized to DAPI staining.

Determination of macrophage content in macrometastatic lesions. Macrophage status of E0771 lung macrometastatic nodules was determined following immunostaining with anti-CD11c (HL3, BD Pharmingen 1:100), rabbit anti-CD206 (ab64693, Abcam 1:100) and rat anti-F4/80 (A3-1, BIO-RAD 1:50) antibodies to detect M1-like TAMs, M2-like TAMs and total TAM population, respectively. M1-like TAM signal was visualized with Alexa Fluor-488 anti-hamster IgG (H+L) (ab173003, Abcam 1:200) secondary antibody while M2-like and total TAMs were detected with Alexa Fluor-488 anti-rabbit IgG (H+L) (A11034, Invitrogen 1:400) secondary antibody. Quantification of M1-like TAMs was defined as the ratio of CD11c and F4/80 overlapping signal to F4/80 positive signal. M2-like TAM was defined as the ratio of CD206 and F4/80 overlapping signal to F4/80 positive signal. Quantification of total F4/80 signal was performed following normalization to DAPI positive area fraction.

Image acquisition. Images of immunolabeled tissue sections were acquired at 10x magnification using the Olympus BX53 fluorescence microscope. We used 4 biological replicates and capture 3 photos per replicate. In order for the images to be comparable they were taken at the same settings and analyzed using a previously developed in-house code in MATLAB (MathWorks, Inc., Natick, MA, USA).

Doxil-induced DNA damage assessment. γH2AX staining was performed to assess Doxil-induced DNA damage in the metastatic nodules of 4T1-implated mice. Mice were orthotopically implanted with 4T1 mouse mammary cancer cells as described earlier and divided into two groups. The first group was orally treated with tranilast (200 mg/kg) once a day from day 4 post-implantation and the second group was given an equal volume of saline until completion of treatment. On day 19, the first group was divided into 2 subgroups of mice, which were treated with saline or Doxil (3mg/kg). Similarly, the second group of mice was divided into 2 subgroups, which received tranilast, or Doxil (3mg/kg) in combination with tranilast (200 mg/kg). Following excision, lungs were fixed in 4% PFA, dehydrated through a series of graded ethanol washes and embedded in paraffin. Transverse 7μm-thick tissue sections were produced

using the microtome (Accu-Cut SRM 200 Rotary Microtome, SAKURA), flatten out into water and allowed to dry overnight at 37°C. Sections were then deparaffinized and subjected to antigen retrieval (microwave heat treatment with TriSodium Citrate, pH 6, for 20 min). Tissue sections were then washed with 1x PBS and incubated in blocking serum (10% Donkey serum, 3% Fetal Bovine Serum, 1x PBS) for 2 hr at room temperature. Next, tissue sections were immunostained with the primary anti-γH2AX antibody (ab22551, Abcam 1:100) and incubated overnight at 4°C. The antibody-antigen interaction was detected using the Mouse Polymer IHC Kit (ab127055, Abcam) according to the manufacture's instructions and finally visualised by adding a DAP chromogen (ab94665, Abcam) that yields a permanent brown precipitate. Following immunostaining, slides were counterstained with hematoxylin, rehydrated and mounted with DPX mountant for histology (Sigma). Immunolabeled slides were scanned at 100x and DNA damage was measured from the ratio of brown positive staining to blue positive fraction.

Collagen deposition in patient specimens using Masson Trichrome. Paraffin embedded lung sections from patients with metastatic breast cancer were deparaffinized and rehydrated as described above. Tissue sections were then stained with Working Weigert's Iron Hematoxylin solution for 5 min, washed and stained with Biebrich Scarlet-Acid Fucshin for 5 min. Finally, slides were rinsed in dH_2O, placed in Phosphotungstic/Phosphomolybdic Acid solution for 5 min and then, in Aniline Blue solution. Next, slides were cleaned in Acetic Acid 1%, rinsed, rehydrated and mounted.

Supporting Information Figures

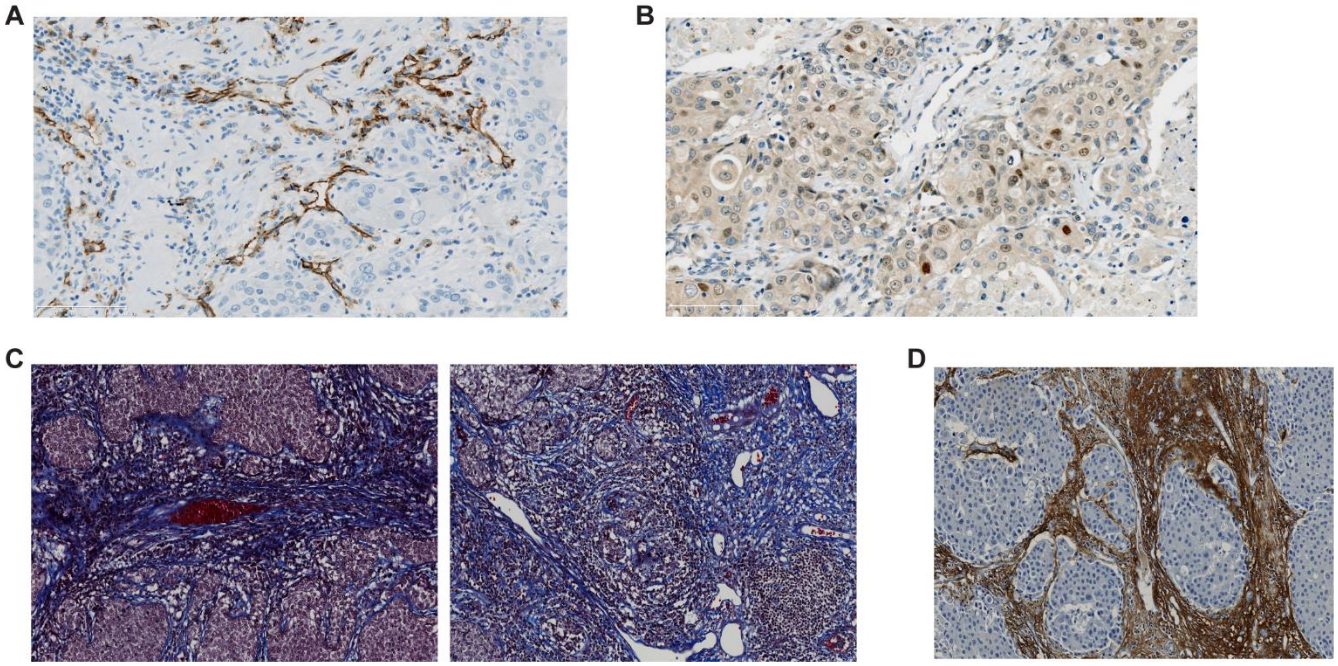

Figure S1. A, Magnified images of CD31 immunostaining reveals irregular vascular proliferation and vessels with small or no lumen, Adapted from. [7] **B,** Magnified images of HIF1α immunostaining reveals weak cytoplasmic staining throughout lesions and occasional intense nuclear staining. Lung metastatic nodules from patients stained with **C,** Masson's trichrome of exhibiting excessive collagen deposition at the sites surrounding blood vessels (blue) and **D,** with anti-hyaluronan binding protein antibodies (brown).

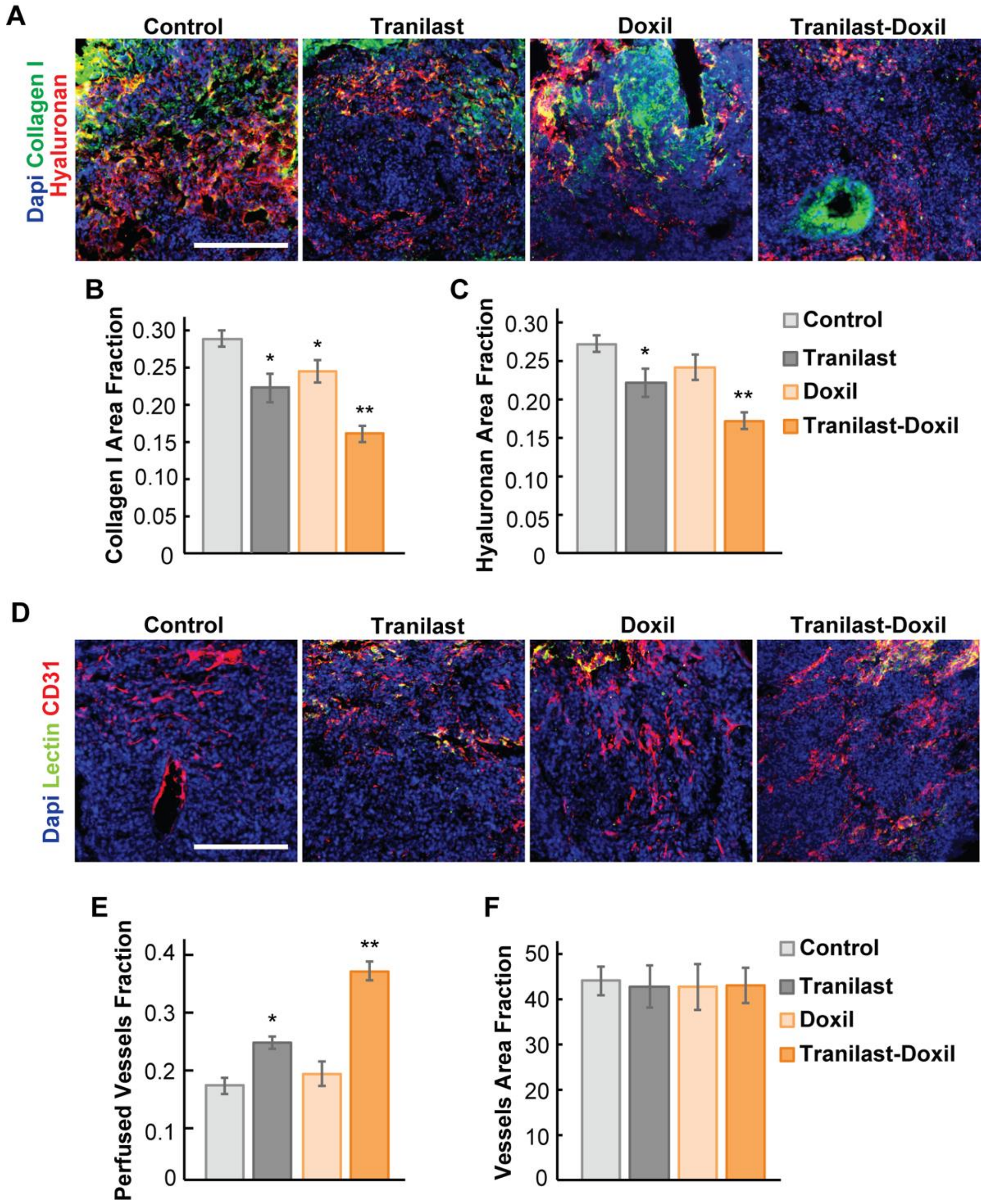

Figure S2. Tranilast-Doxil normalizes tumor microenvironment and improves perfusion of E0771 metastatic nodules. **A,** Representative fluorescence images of collagen I (green), hyaluronan (red) immunostaining and DAPI nuclear staining of E0771 metastatic nodules treated as indicated. **B,** Quantification of collagen I and hyaluronan **(C)** area fraction in the interstitial microenvironment of lung nodules. **D,** Representative fluorescence images of E0771 metastatic nodules immunostained for biotinylated tomato lectin (green), CD31 (red) and DAPI (blue) after various treatments. **E,**

Quantification of vascular perfusion indicated by the co-expression of CD31 endothelial marker and lectin (yellow). **F,** Vessel fraction as defined by CD31 (red) positive staining. Statistical analyses were performed by comparing the treated groups with the control * and the tranilast-Doxil group with all other treatment groups **, $p<0.05$, (n=8-10). Scale bar: 200μm.

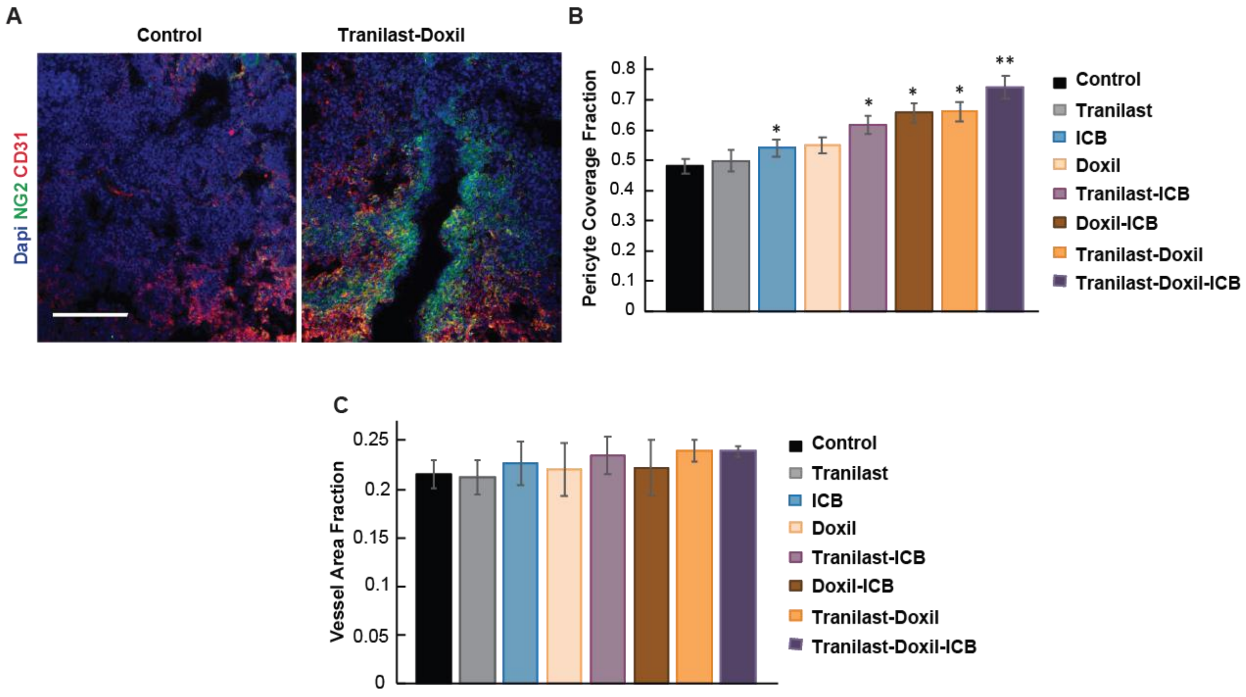

Figure S3. Pericyte coverage quantification in 4T1 lung metastases as a measure of normalization of the tumor vessel wall. Mice bearing 4T1 tumors were treated with tranilast, Doxil, a cocktail of anti-PD1 and anti-CTLA-4 antibodies for immune checkpoint blockade (ICB) or a combination of them. **A,** Representative immunofluorescence images of 4T1 lung metastatic lesions obtained from control and tranilast-Doxil treated mice stained with CD31 (red) endothelial marker, NG2 (green) pericyte marker and DAPI (blue, cell nuclei). **B,** Blood vessel pericyte coverage was determined by the colocalization of CD31 and NG2 positive staining (yellow) normalized to total CD31 area fraction. **C**, The number of blood vessels remains the same following different treatments as indicated by the quantification of CD31 area fraction in 4T1 lung metastasis. Statistical analyses were performed by comparing the treated groups with the control *, $p<0.05$, and the tranilast-Doxil-ICB group with all other treatment groups **, $p<0.05$, (n=8-10). Scale bar: 200μm.

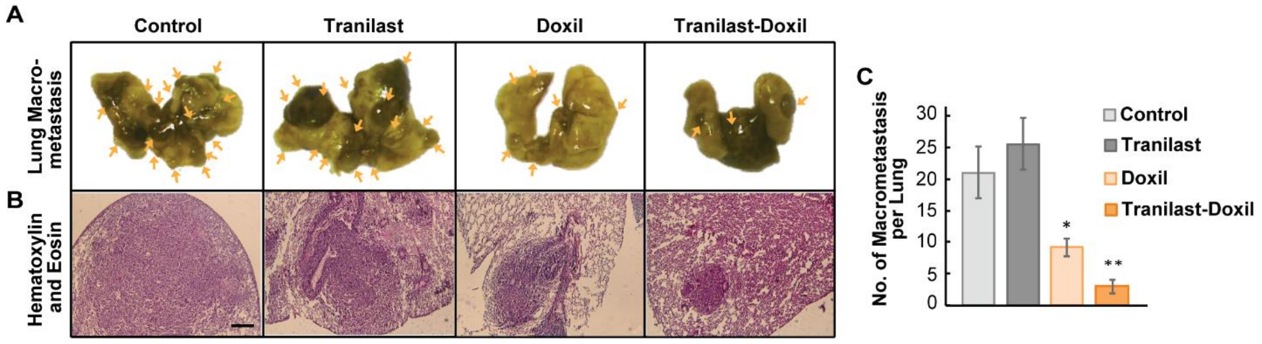

Figure S4. Tranilast-Doxil treatment reduces lung metastasis of E0771 primary breast tumors. **A,** Lung photographs of E0771 murine breast tumors treated as indicated. Yellow arrows indicate the macrometastatic nodules on the lungs. **B,** Representative H&E histopathological images of lungs bearing E0771 tumors. **C,** Quantification of nodules counted in both sides of lung (front and back) under a stereoscopic microscope after treated as indicated. Statistical analyses were performed by comparing the treated groups with the control * and the tranilast-Doxil group with all other treatment groups **, $p<0.05$, (n=8-10). Scale bar: 200μm.

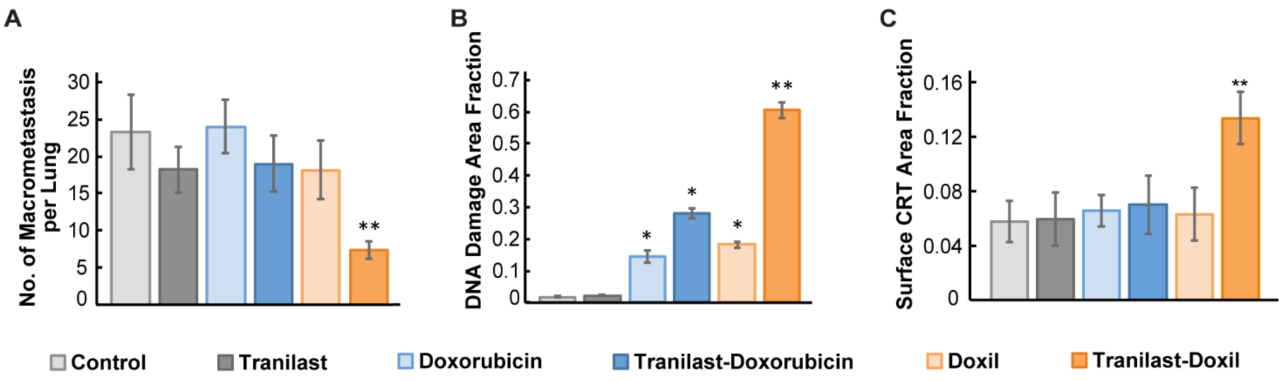

Figure S5. Combinatorial tranilast-doxorubicin fails to induce similar to tranilast-Doxil effects. The bar graphs are the same as in Figure 3 of the main text and incorporates data for two additional groups: doxorubicin and tranilast-doxorubicin. Doxorubicin was administered at a dose of 5mg/kg, intraperitoneally every 3 days, following our previous studies [3, 8]. Tranilast-doxorubicin fails to reduce 4T1 metastasis (**A**) and immunogenic cell death (**C**), whereas only moderately increases DNA damage (**B**). These superior effects of the use of Doxil-nanomedicine compared to the conventional chemotherapy are in agreement with our previous study [8]. Statistical analyses were performed by comparing the treated groups with the control * and the tranilast-Doxil group with all other treatment groups **, $p<0.05$, (n=8-10).

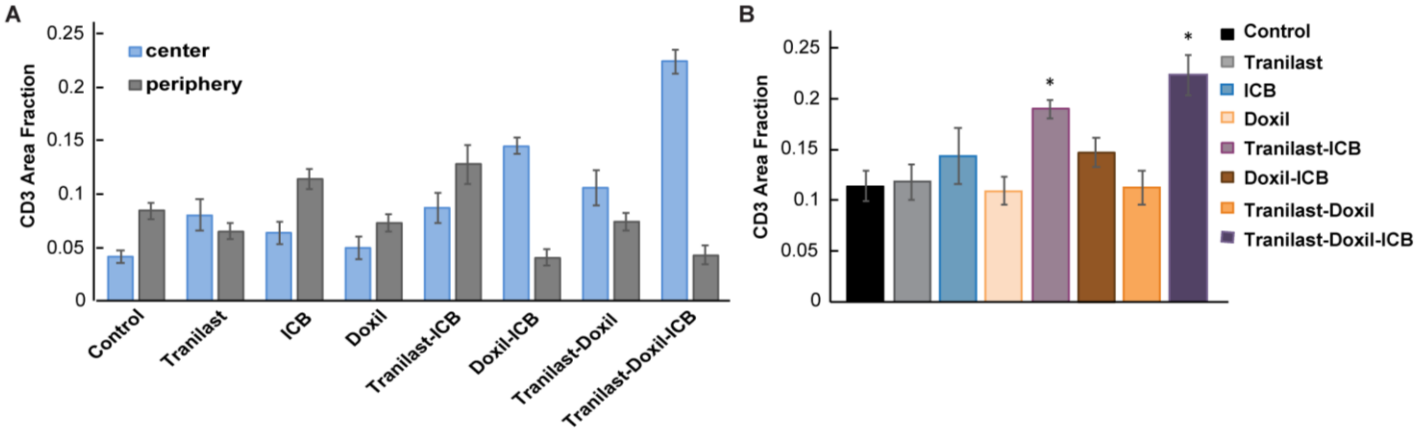

Figure S6. **A,** Quantification of peripheral and central CD3 positive staining in the metastatic lesions of 4T1 tumors treated as indicated. **B,** Total T cell population increases following treatment with tranilast-ICB and tranilast-Doxil-ICB. Statistical analyses were performed by comparing the treated groups with the control *, $p<0.05$, (n=8-10).

References

1 M. Hiroi, M. Onda, E. Uchida, and T. Aimoto, *J Nippon Med Sch*. 69, 224-34 (2002)

2 R. Chakrabarti, V. Subramaniam, S. Abdalla, S. Jothy, and G. J. Prud'homme, *Anticancer Drugs*. 20, 334-45 (2009)

3 P. Papageorgis, C. Polydorou, F. Mpekris, C. Voutouri, E. Agathokleous, C. P. Kapnissi-Christodoulou, and T. Stylianopoulos, *Sci Rep*. 7, 46140 (2017)

4 V. P. Chauhan, J. D. Martin, H. Liu, D. A. Lacorre, S. R. Jain, S. V. Kozin, T. Stylianopoulos, A. Mousa, X. Han, P. Adstamongkonkul, Z. Popovic, M. G. Bawendi, Y. Boucher, and R. K. Jain, *Nature Communications*. 4, 10.1038/ncomms.3516 (2013)

5 F. Mpekris, P. Papageorgis, C. Polydorou, C. Voutouri, M. Kalli, A. P. Pirentis, and T. Stylianopoulos, *J Control Release*. 261, 105-112 (2017)

6 C. Polydorou, F. Mpekris, P. Papageorgis, C. Voutouri, and T. Stylianopoulos, *Oncotarget*. 8, 24506-24517 (2017)

7 J. D. Martin, M. Panagi, C. Wang, T. T. Khan, M. R. Martin, C. Voutouri, K. Toh, P. Papageorgis, F. Mpekris, C. Polydorou, G. Ishii, S. Takahashi, N. Gotohda, T. Suzuki, M. E. Wilhelm, V. A. Melo, S. Quader, J. Norimatsu, R. M. Lanning, M. Kojima, M. D. Stuber, T. Stylianopoulos, K. Kataoka, and H. Cabral, *ACS Nano*. 13, 6396-6408 (2019)

8 M. Panagi, C. Voutouri, F. Mpekris, P. Papageorgis, M. R. Martin, J. D. Martin, P. Demetriou, C. Pierides, C. Polydorou, A. Stylianou, M. Louca, L. Koumas, P. Costeas, K. Kataoka, H. Cabral, and T. Stylianopoulos, *Theranostics*. 10, 1910-1922 (2020)
